# 

**Published:** 1902-12-13

**Authors:** 


NOTICE TO CORRESPONDENTS.

All MSS., Letters, Books for Review, and other matters intended for the Editor should be addressed The Editor, "The Hospital," The Hospital Building, 28 & 29 Southampton Street, Strand, W.C.

The Editor cannot undertake to return rejected MS., even when accompanied by stamped directed envelope.

Notice to Advertisers and Subscribers.

All Advertisements, Orders for Copies of The Hospital, and Business Communications should be addressed to THE MANAGER, (Not to the Editor), The Hospital, 28 & 29 Southampton Street, Strand, London, W.C.

The Cost of Subscription, post free, is as follows.—Per week, 2½d.; per quarter, 2s. 8d.; per half-year, 5s. 3d.; per annum, 10s. 6d. Foreign:—Per annum, 15s. 2d., payable, in all cases, in advance.

NOTICE.—Vol. XXXII. of "The Hospital" (April to September 1902) in handsome cloth binding is now on sale at the office of this Journal, price, 7s. 6d. Cases for binding the Numbers contained in this Volume 1s. 6d. Index to Vol. XXXII., 2½d., post free.

The Monthly Part for December is now ready. Price 6d., by post 8d.

### BOOKS RECEIVED.

C. Griffin and Co.

"Official Year Book of the Scientific and Learned Societies of Great Britain and Ireland, 1902."

McCorquodale and Co., Limited.

"Metropolitan Asylums Board Annual Report, 1901." 2 vols.

J. B. Lippincott Co.

"International Clinics." Vol. iii. Twelfth series.

The Army and Navy Gazette.

"Mentioned in Despatches." Complete list. Second Edition.

Baillière, Tindall and Cox.

"Operative and Clinical Surgery." By W. Stokes. 10s. net.

R. L. Sharland.

"The Scientific Roll 'Bacteria.'" Vol. i, No. 6.

The Scientific Press.

"The Nursing Profession." Fourth year. By Sir Henry Burdett, K.C.B. 2s. net.

### The Student of Medicine.

### APPOINTMENTS.

**H. J. Bryan**, M.R.C.S., L.R.C.P.Lond., appointed District Medical Officer of the Medway Union. **Miss I. S. Bryson**, M.B.Lond., appointed Assistant Medical Officer to the Camberwell Parish Infirmary. **F. Burghard**, M.B., F.R.C.S., appointed Teacher of Operative Surgery at King's College, London. **A. Carless**, M.B., F.R.C.S., appointed Professor of Surgery at King's College, London. **W. Watson Cheyne**, C.B., F.R.S., F.R.C.S., appointed Professor of Clinical Surgery at King's College, London. **M. Farrant, jun.**, M.R.C.S.. L.R.C.P.Lond., appointed District Medical Officer of the St. Thomas's Union. **T. Barrett Heggs**, M.B., Ch.B.Aberd., appointed House Surgeon at the Sussex County Hospital, Brighton. **Francis J. Joynes**, M.R.C.S.Eng., reappointed Medical Officer of Health to the Dursley Rural District Council. **J. S. Macdonald**, L.R.C.P.&S.Edin., appointed Professor of Physiology at Sheffield University College. **J. G. MacNaughton**, M.D., C.M., M.R.C.P.E., appointed Pathologist and Assistant Medical Officer to the Hospital for Consumption and Diseases of the Throat and Chest, Manchester. **T. Pettey**, M.D.Edin., F.R.C.S Eng., appointed Medical Officer to the First District of the Eastbourne Union. **Arthur C. Roper**, F.R.C.S.Edin., appointed Surgeon to the West of England Eye Infirmary, Exeter. **James Sherrew**, F.R.C.S.Eng., appointed Assistant Demonstrator of Anatomy at the London Hospital Medical College and Assistant Surgeon to the London Hospital. **B. W. Swenden**, M.R.C.S., L.R.C.P.Lond., appointed Medical Officer for the West and North-West Districts of the Darlington Union. **J. Terry**, M.R.C S., L.R.C.P.Lond., appointed District Medical Officer of the Daventry Union. **W. J. Woodman**, M.R.C.S., L.R C.P.Lond., appointed District Medical Officer of the Medway Union.

St. Thomas's Hospital.—The following gentlemen have been selected as House Officers from Tuesday, December 2nd, 1902.—House Physicians: **W. H. Harwood Yarred**, B.Sc.Lond., L.R.C.P., M.R.C S.; **A. Mavrogordato**, L.R.C.P., M.R.C.S. Assistant House Physicians: **C. N. Sears**, L.R.C.P., M.R.C.S.; **A. E. Boycott**, M.A., M.B., B.Ch.Oxon, B.Sc.Oxon. Obstetric House Physicians: (*Senior*) **F. J. Child**, M.A., M.B., B.C.Cantab., L.R.C.P, M.R.C.S.; (*Junior*) **G. A. C. Shipman**, M.A., M.B., B.C.Cantab., L.R.C.P., M.R.C.S. Clinical Assistants in the Special Department for Diseases of the Ear: **B. S. Jones**, L.R.C.P., M.R.C.S.; **S. G. Scott**, M.A., M.B, B.Ch.Oxon.

Several other gentlemen have received extensions of their appointments.

**"The Hospital" Institutional Library.**

"Hospitals and Asylums of the World." 4 Vols., with a Portfolio of Plans, complete £12 12s.

"Burdett's Hospitals and Charities, 1902." 5s.

"Cottage Hospitals, General, Fever, and Convalescent." 10s. 6d.

"Hospital Expenditure: The Commissariat." 2s. 6d.

"A Legal Handbook for the Use of Hospital Authorities." 2s. 6d.

"The Cottage Hospital Case Book or Register of Patients." 10s. and 12s. 6d.

"The Furnishing and Appliances of a Cottage Hospital." 6d.

All these are published by the Scientific Press, Ltd., and may be obtained through any bookseller, or direct from the publishers, 28 and 29 Southampton Street, Strand, London, W.C.

